# A baseline assessment of antimicrobial stewardship core element implementation in selected public hospitals in Malawi: findings from the 2023 National Program Audit

**DOI:** 10.3389/fpubh.2025.1588778

**Published:** 2025-06-12

**Authors:** Ronald Chitatanga, Chikhulupiliro Yiwombe, Oscar Divala, Mwayi Prudence Msokera, Ellen Banda, Hope Chadwala, Manuel Wellington Gilmon, Wezi Kaminyoghe, Innocent Chibwe, Harry Milala, Alinafe Kawerama, Kenneth Nyoni, Chikumbutso Mpanga, Christina Mwinjiwa, Akuzike Makondesa, Maurice Mwehiwa, Donald Mlombwa, Daniel Namalawe, Silver Benito, Emmie Jingini, Evelyn Wesangula, Martin Matu, Emily Ciccone, Robert Krysiak, Collins Mitambo, Titha Dzowela

**Affiliations:** ^1^Antimicrobial Resistance Coordinating Centre, Public Health Institute of Malawi, Lilongwe, Malawi; ^2^Fleming Fund Country Grant, University of North Carolina Project-Malawi, Lilongwe, Malawi; ^3^Antimicrobial Stewardship Committee, Kamuzu Central Hospital, Lilongwe, Malawi; ^4^Antimicrobial Stewardship Committee, Mzimba South District Hospital, Mzimba, Malawi; ^5^Antimicrobial Stewardship Committee, Queen Elizabeth Central Hospital, Blantyre, Malawi; ^6^Antimicrobial Stewardship Committee, Malamulo Adventist Hospital, Thyolo, Malawi; ^7^Antimicrobial Stewardship Committee, Zomba Central Hospital, Zomba, Malawi; ^8^Antimicrobial Stewardship Committee, Mzuzu Central Hospital, Mzuzu, Malawi; ^9^ECSA Secretariat, East, Central, and Southern Africa Health Community, Arusha, Tanzania; ^10^Fleming Fund Country Grant, University of North Carolina at Chapel Hill, Chapel Hill, NC, United States

**Keywords:** antimicrobial stewardship, antimicrobial resistance, public hospitals, core elements, Malawi

## Abstract

**Background:**

Antimicrobial resistance (AMR) is a significant global health challenge, particularly in low- and middle-income countries (LMICs). In Malawi, frequent stockouts of essential medicines and the widespread dispensing of antibiotics without prescriptions have exacerbated the AMR burden, highlighting the urgent need for robust antimicrobial stewardship (AMS) interventions. This study presents the first documented baseline assessment of AMS core elements across six public healthcare facilities within Malawi’s AMR sentinel surveillance network. Understanding the baseline status of AMS implementation provides a critical reference point to guide future interventions, inform policy, and prioritize resources in the national response to AMR.

**Materials and methods:**

This descriptive analysis used data from a national AMS program audit conducted from July 10–14, 2023, in six public hospitals: Malamulo Adventist Hospital, Mzimba South District Hospital, Kamuzu Central Hospital, Queen Elizabeth Central Hospital, Zomba Central Hospital, and Mzuzu Central Hospital. The World Health Organization (WHO) Healthcare Facility AMS Assessment Tool was used to evaluate implementation across key AMS domains, including leadership, accountability, stewardship actions, education, monitoring, surveillance, and reporting. A total of 30 AMS committee members participated using a consensus-based approach.

**Results:**

Of the six hospitals assessed, only one (Kamuzu Central Hospital) demonstrated strong implementation of AMS core elements, achieving a score of 79%. The remaining facilities reported moderate to low performance, with Mzimba District Hospital scoring the lowest (24%). Leadership commitment was inconsistent; only one (16.7%) hospital had fully integrated AMS into its annual plans, and resource allocation was limited. AMS ward rounds and antibiotic prescription audits were either absent or only partially implemented across most facilities. Education and training initiatives were fragmented, with only one (16.7%) hospital partially integrating AMS into staff induction.

**Conclusion:**

This situational analysis reveals critical gaps in AMS implementation across Malawi’s national AMR surveillance hospitals. Limited leadership commitment, infrequent AMS ward rounds, and inconsistent education for healthcare workers were major barriers. Targeted interventions are needed to strengthen leadership, establish feasible facility-level AMS actions, and build sustainable capacity among healthcare workers.

## Introduction

Infections have become significantly more treatable, in recent decades, with the discovery and widespread use of antimicrobial agents. However, antimicrobial resistance (AMR) has quickly increased due to their overuse in veterinary and human medicine (1). This increase in AMR seriously threatens the health and welfare of people, animals and plants (1). The World Health Organization (WHO) has identified AMR as one of the top 10 priority global challenges, warning of a looming post-antimicrobial era where common infections and minor injuries could once again become fatal (2). The emergence of multidrug-resistant microbes has rendered many previously effective treatments ineffective, leading to longer hospital stays, higher medical costs and increased mortality rates (3). According to literature, more than 1.14 million (95% UI: 1·00–1·28) human deaths were attributed to resistant bacterial infections, with projections indicating that these deaths could reach 10 million in 2050 if AMR is not addressed (4, 5).

Antimicrobial resistance is increasing, especially in low- and middle-income countries (LMICs) compared to high-income countries. This is mostly due to the overuse and misuse of antibiotics in healthcare (6). The burden of AMR is worse in sub-Saharan Africa, particularly in Malawi, where there is a low adherence to national standard treatment guidelines, limited antibiotic alternatives and frequent stockouts (7). To address this growing crisis, the implementation of antimicrobial stewardship (AMS) is crucial. AMS involves a coordinated set of actions that promote responsible antibiotic use at both the hospital and community settings (8). Hospital AMS programs have been demonstrated to improve patient outcomes while optimising antibiotic use and reducing bacterial resistance (9).

Following WHO guidance on how to establish, implement and evaluate AMS systems (8), Malawi launched its national AMS program in 2023, with structured guidelines targeting a network of seven public healthcare facilities that form the national AMR sentinel surveillance network. This network included four tertiary-level facilities (Mzuzu Central Hospital, Kamuzu Central Hospital, Zomba Central Hospital and Queen Elizabeth Central Hospital), two secondary-level facilities (Mzimba South District Hospital and Mangochi District Hospital) and one faith-based hospital (Malamulo Adventist hospital). These strategically selected sites have standard laboratory capacity to support AMR detection and surveillance (10).

At the commencement of the AMS program, the Ministry of Health conducted a baseline evaluation of AMS core elements within these facilities to understand the existing capacities and system-level gaps—an approach aligned with practices observed in other low- and middle-income countries across Africa, Asia, and Europe (11). This study analyses data from that baseline audit to document the initial state of AMS implementation in Malawi, providing a reference point for tracking future progress and informing targeted AMS interventions nationally.

Considering the paucity of information regarding the level of implementation of WHO healthcare facility AMS core elements, which include leadership commitment, accountability, antimicrobial stewardship actions, monitoring and surveillance, education and training of AMS teams and reporting to prescribers, this study documents baseline assessment of the implementation of AMS in selected Malawian sentinel AMR surveillance healthcare facilities. The WHO healthcare Facility AMS assessment tool (8), which was specifically designed for healthcare facilities in LMICs, was used in the audit. Therefore, we provide a reference point for quality improvement in the national AMS program.

## Materials and methods

### Study design

This was a descriptive cross-sectional analysis of data from a Ministry of Health audit of the national AMS program conducted in 2023. This audit involved healthcare facilities in the national AMR surveillance network, aiming to assess the implementation of AMS in selected healthcare facilities in Malawi. This audit was conducted between 10 and 14 July 2023, and was led by the National Antimicrobial Resistance Coordinating Center (AMRCC) of the Malawi Ministry of Health. Technical support and funding were provided by the East, Central and Southern Africa Health Community (ECSA-HC).

In the audit, a WHO Healthcare Facility AMS Assessment tool (8) was administered as a questionnaire to selected AMS committee members in eligible hospitals. This tool was previously validated in LMIC contexts, including Malawi, through a multi-country feasibility study (12) which demonstrated the toolkit’s acceptability, feasibility and utility in guiding AMS program development. This questionnaire was completed through a group consensus-based approach, whereby members of each facility’s AMS committee collaboratively discussed and responded to each item in the tool. This method ensured that inputs reflected the collective institutional experience and practices rather than the opinion of a single respondent. Respondents were guided through each question by a trained facilitator, and final responses were agreed upon only after deliberation and consensus among all participants.

### Study setting and sampling frame

This audit targeted healthcare facilities that were, at the time of the assessment, actively participating in Malawi’s national AMR sentinel surveillance network. The network comprised seven strategically selected hospitals across the country, including four tertiary-level facilities—Mzuzu Central Hospital (Northern Region), Kamuzu Central Hospital (Central Region), Zomba Central Hospital, and Queen Elizabeth Central Hospital (both in the Southern Region). It also included two secondary-level hospitals: Mzimba South District Hospital (Northern Region) and Mangochi District Hospital (Southern Region), as well as Malamulo Adventist Hospital, a faith-based institution providing secondary-level care in the Southern Region.

To be eligible for inclusion in the 2023 AMS, each facility was required to have a formally instituted and functional AMS committee that had been operational for at least 3 months prior to the assessment. All tertiary level hospitals had functional AMS programs for 6 months while the other facilities had 3 months of implementation prior to the audit. Facilities not affiliated with the AMR surveillance network or lacking an active AMS program were excluded from the audit.

In each healthcare facility eligible for the AMS audit, a purposive sampling technique was employed to select respondents. This ensured representation of diverse professional roles within each AMS committee. Five members were purposefully selected from each committee based on their professions, which included a medical doctor, pharmacist, nurse, laboratory professional, and representative from hospital management. This approach captured a collective and multidisciplinary perspective of AMS practices within each facility.

### Data collection

The structured questionnaire version of the WHO Healthcare Facility AMS Assessment tool (8) was administered in person through a paper-based format and responses digitised using KoboToolbox, a free open-source tool for mobile data collection (13). This questionnaire (Supplementary file) consisted of sections which included questions on the AMS core elements. A total of 41 questions related to the AMS core elements were incorporated in the questionnaire, these were divided as follows: 7 questions on leadership commitment, 10 on accountability, 11 on antimicrobial stewardship actions, 6 on monitoring and surveillance, 3 on education and training, and 4 on reporting to prescribers. Each question was rated on a five-point scale: ‘No’, ‘No, but a priority’, ‘Planned but not started’, ‘Partially implemented’ and ‘Yes, fully implemented’. Trained facilitators guided each healthcare facility team in filling the questionnaire.

### Data analysis

All data analysis in this study was performed using R statistical software (version 4.3.3) (14). Firstly, the dataset was exported from KoboToolbox in XLS format and then data cleaning was conducted. During data cleaning, the dataset was reviewed for consistency, with duplicate entries and data entry errors corrected prior to analysis. Each AMS core element question, structured as a 5-point ordinal scale—(1) No, (2) No but a priority, (3) Planned but not started, (4) Partially implemented, and (5) Fully implemented—was assigned a numeric value from 0 to 4. This coding allowed the transformation of qualitative implementation status into quantitative summary scores, enabling facility-level comparison.

Descriptive analysis focused on six key AMS core components: leadership commitment, accountability, AMS actions, monitoring and surveillance, education and training, and reporting (8). To facilitate comparative interpretation, implementation scores for each question were aggregated at the theme level and visualized using heatmaps, with a color gradient representing the degree of implementation across facilities. These heatmaps highlighted thematic strengths and weaknesses within and between hospitals, offering a visual overview of AMS progress.

In addition to thematic visualization, a composite AMS score was calculated for each facility by summing all individual question scores and dividing by the maximum possible score, then expressing this as a percentage. This score offered a simplified metric for benchmarking AMS implementation. Facilities were ranked based on their total scores, and results were tabulated for clarity.

All data manipulation and visualization were conducted using key R packages: “tidyverse” for data cleaning and wrangling, “ggplot2” for visualization, and “flextable” for publication-ready tables. The dataset are available in a public repository for further validation.^1^

Finally, this study adhered to Strengthening the Reporting of Observational studies in Epidemiology (STROBE) guidelines for cross-sectional studies in reporting observational health system data (15).

### Ethical considerations

The antimicrobial stewardship audit conducted by the Antimicrobial Resistance Coordinating Centre of the Malawi Ministry of Health did not require ethical approval, as it involved the collection of routine health system data from the national AMS program as part of ongoing program monitoring. This audit is conducted annually to assess progress in AMS implementation across healthcare facilities. Additionally, ethical review is not warranted because this audit poses no direct risk to human subjects and does not involve the collection of personal health data, patient records, or any other sensitive information. Prior verbal permission to engage AMS committees in each facility was obtained from hospital leadership.

However, approval to publish and disseminate the findings was obtained from the National Health Sciences Research Committee (NHSRC) in Malawi (#: 06/12/2024). This ensured full compliance with ethical and regulatory standards.

## Results

**Figure 1:**
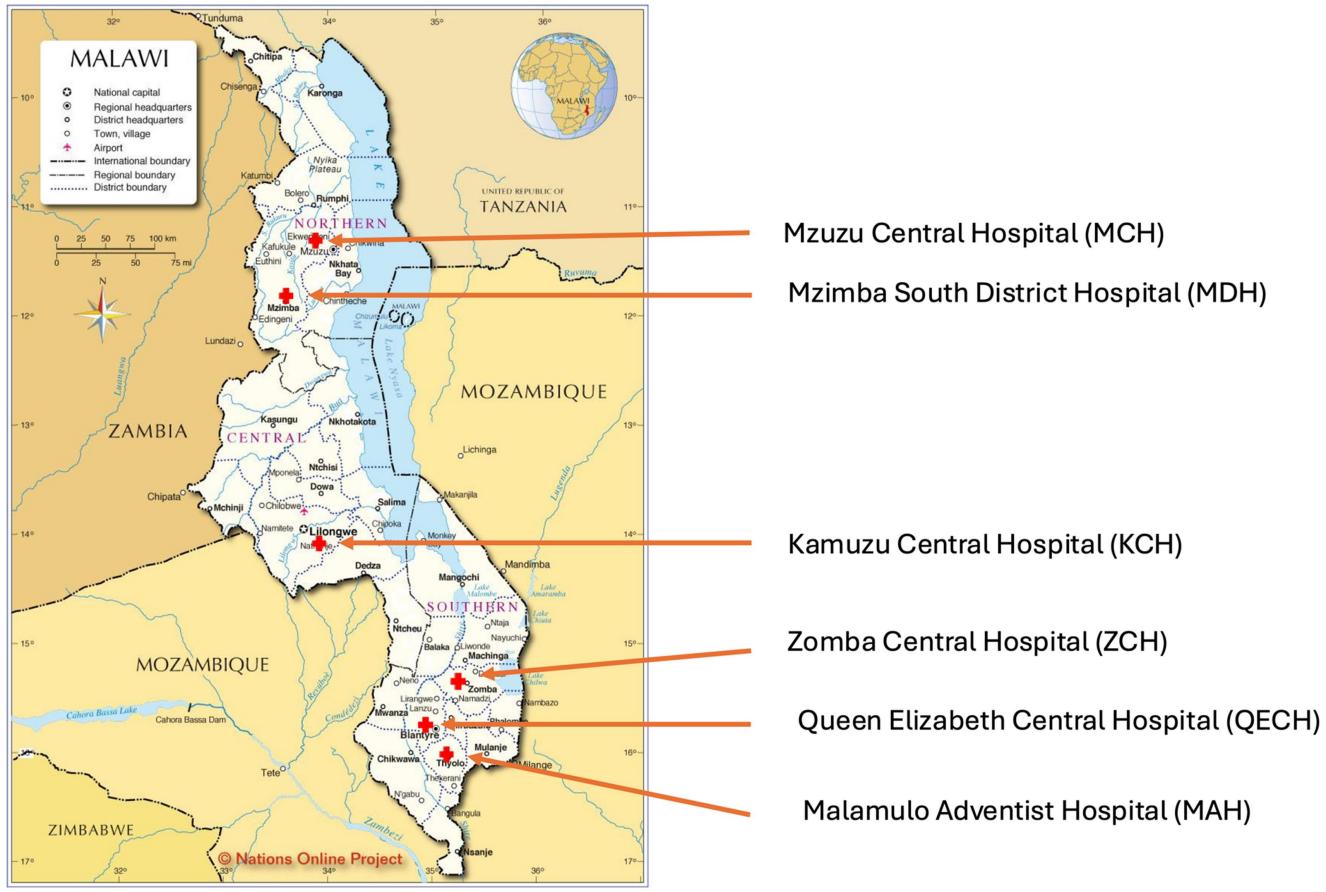
Map of Malawi (26) showing the audited healthcare facilities.

Data from six of the seven hospitals (Figure 1) in the national AMR surveillance network that participated in the AMS audit were analysed. These hospitals were Mzuzu Central Hospital (MCH), Kamuzu Central Hospital (KCH), Zomba Central Hospital (ZCH), Queen Elizabeth Central Hospital (QECH), Malamulo Adventist Hospital (MAH), and Mzimba South District Hospital (MDH). A total of 30 participants contributed to the assessment, comprising five healthcare workers from each hospital who provided collective insights on AMS implementation.

### Leadership commitment to antimicrobial stewardship

**Figure 2:**
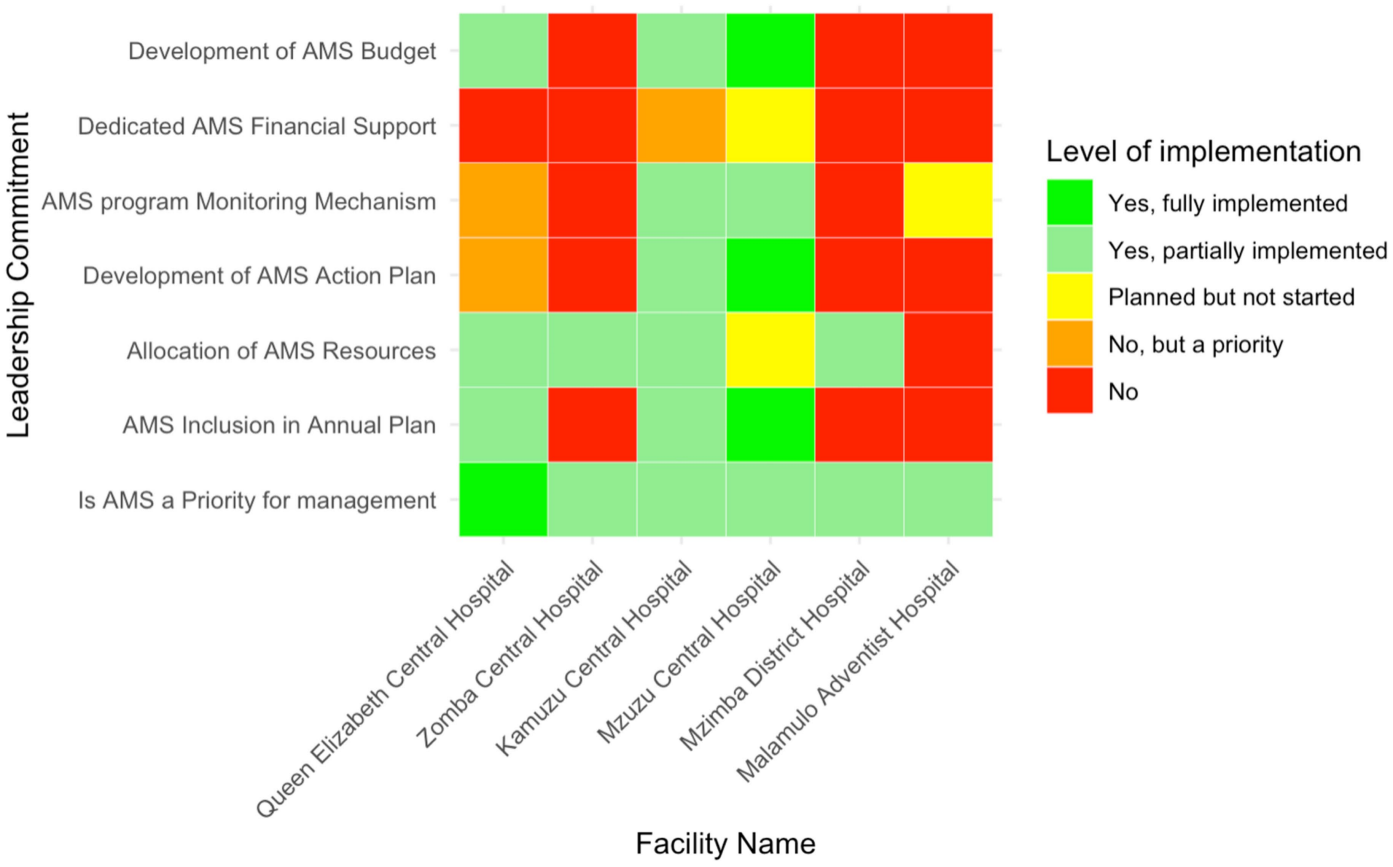
Heatmap of leadership commitment for AMS implementation in six Malawian hospitals. The color gradient reflects the range from no implementation (red) to full implementation (green).

Aspects of commitment to AMS by hospital leadership are shown in Figure 2. Notably, AMS was prioritised by hospital management in all audited facilities. One facility, Queen Elizabeth Central Hospital—representing 16.7% (*n* = 1/6) of the facilities—reported full implementation of AMS as a core priority, while the remaining five hospitals had reported a partially implemented facility AMS program. Integration of AMS into annual operational plans varied significantly, where three hospitals, representing 50% (*n* = 3/6) of the total—Zomba Central Hospital, Mzimba District Hospital and Malamulo Adventist Hospital—did not include AMS in their annual plans. Mzuzu Central Hospital was the only facility to fully integrate AMS into its plans, whereas Kamuzu Central Hospital and QECH partially incorporated AMS.

Resource allocation for AMS activities also varied, with four hospitals (66.7%; *n* = 4/6)—MDH, KCH, ZCH, and QECH— reporting to partially allocate resources, which included human resources, while MAH reported not to allocate any resources. MCH had planned to allocate resources but had not yet begun. AMS monitoring mechanisms were partially established in MCH and KCH, while MDH and ZCH lacked these systems. Furthermore, financial support for AMS activities was limited, with only MCH (16.7%, *n* = 1/6) having planned efforts to secure funding, while MAH, MDH and ZCH had no AMS budgets.

### Accountability in antimicrobial stewardship programs

**Figure 3:**
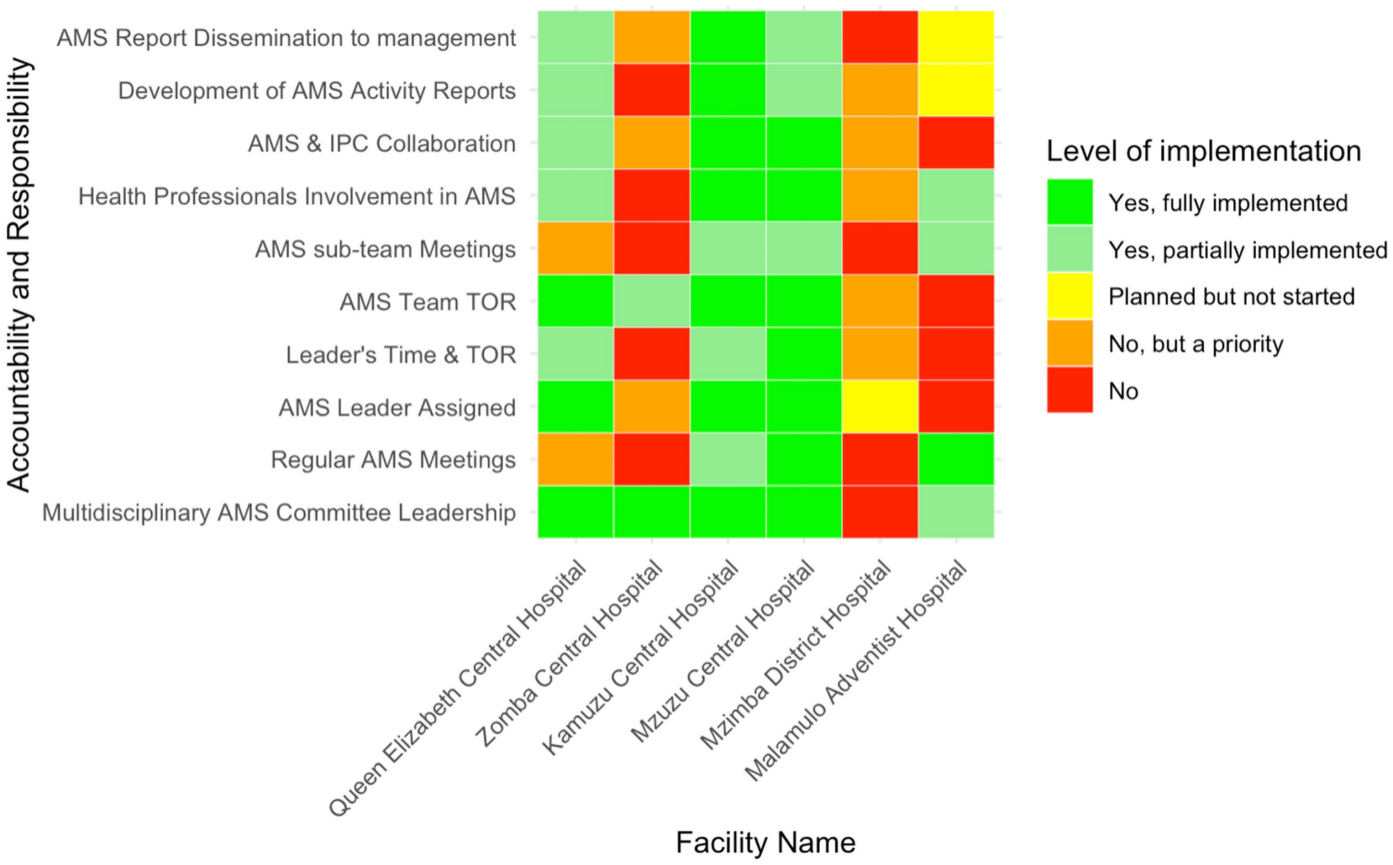
Heatmap of the level of implementation of AMS accountability and responsibility in six Malawian hospitals.

The level of accountability and responsibility in AMS implementation varied significantly across the six facilities (Figure 3). Multidisciplinary AMS committee leadership was reportedly fully implemented in all the tertiary-level hospitals, while Mzimba District Hospital lacked such leadership. Regular AMS committee meetings were only consistently held in two facilities (33.3%, *n* = 2/6), Malamulo Adventist Hospital and Mzuzu Central Hospital.

Half of the hospitals had designated AMS leaders, but MAH, MDH and ZCH had not yet assigned this responsibility. The implementation of terms of reference (TOR) for AMS leaders and teams showed partial adherence, with KCH and QECH reporting partially implemented TORs for leaders, while ZCH partially implemented them for teams. Multisectoral collaboration was fully realised only in MCH and KCH—representing 33.3% (*n* = 2/6)—while QECH reported partial collaboration and the other hospitals did not engage other sectors. Regarding AMS activity reporting, KCH fully implemented these processes, while MAH was still in the planning phase and MDH acknowledged the need but had not prioritised these activities.

### Implementation of AMS actions across facilities

**Figure 4:**
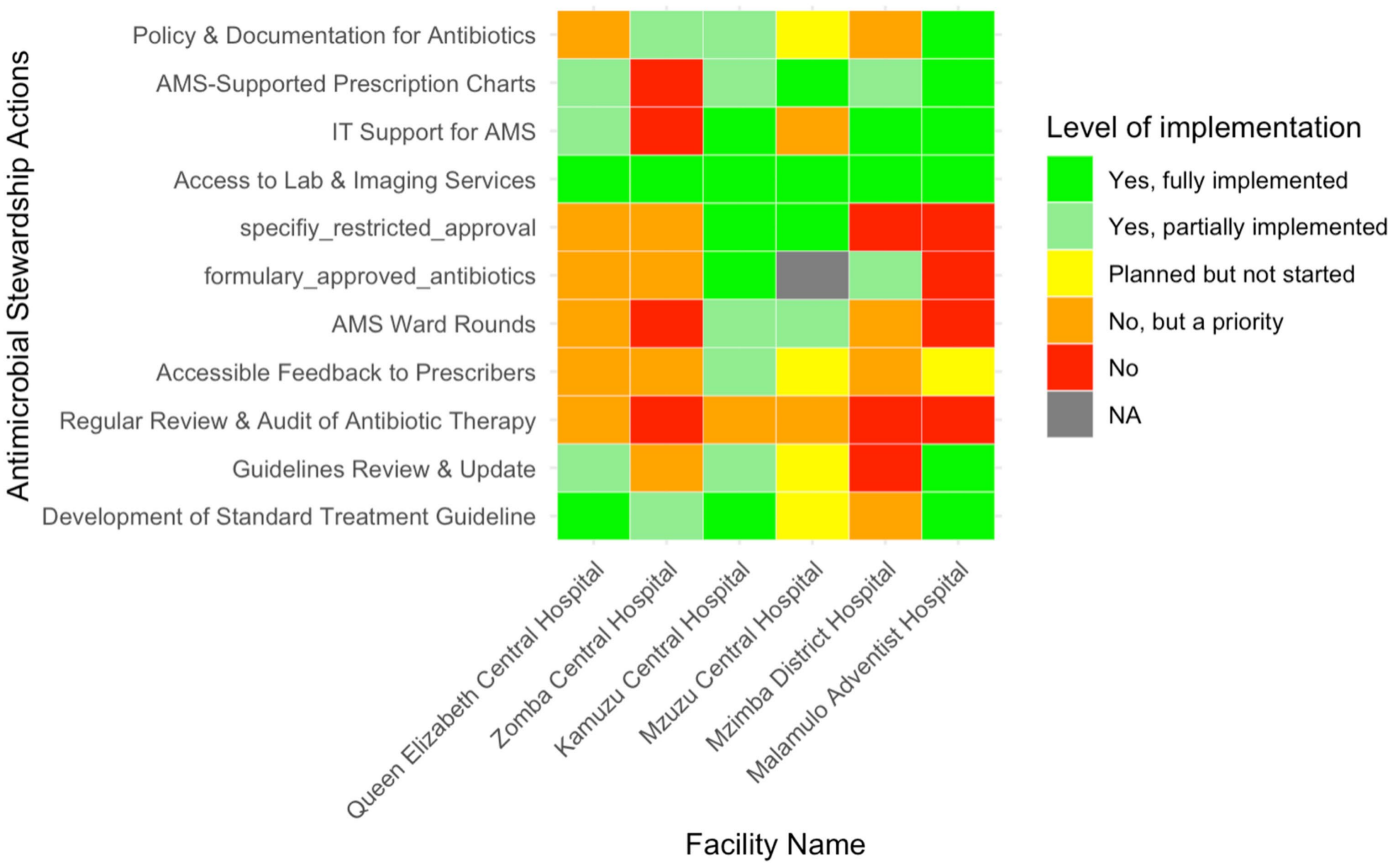
Heatmap illustrating the level of implementation of various Antimicrobial Stewardship actions in six Malawian hospitals.

AMS actions included specific interventions undertaken by the hospital AMS team to rationalise antibiotics. Across the six facilities, three (50%, *n* = 3/6) (Queen Elizabeth Central Hospital, Kamuzu Central Hospital, and Malamulo Adventist Hospital) reported fully implemented use of standard treatment guidelines, while Zomba Central Hospital reported partial implemention (Figure 4). Mzimba District Hospital, although recognising the need for these guidelines, had not yet initiated implementation. Updates and reviews of these guidelines were more inconsistent, with only Malamulo Adventist Hospital fully implementing this process. Queen Elizabeth Central and Kamuzu Central Hospitals had partially updated their guidelines, whereas Zomba Central had not yet commenced any reviews, despite acknowledging their necessity.

Antibiotic therapy audits and feedback mechanisms to prescribers were lacking in most facilities, with only partial implementation seen at Kamuzu Central, while Malamulo Adventist and Mzuzu Central Hospitals had reportedly only planned these mechanisms. AMS ward rounds were also partially implemented in Mzuzu and Kamuzu Central Hospitals. The development of a formulary for approved antibiotics was fully implemented at Kamuzu Central, with Mzimba District Hospital only partially meeting this goal. All hospitals had full access to laboratory and imaging services, and IT support for AMS was fully implemented in three hospitals (50%, *n* = 3/6).

### Educational initiatives within AMS programs

**Figure 5:**
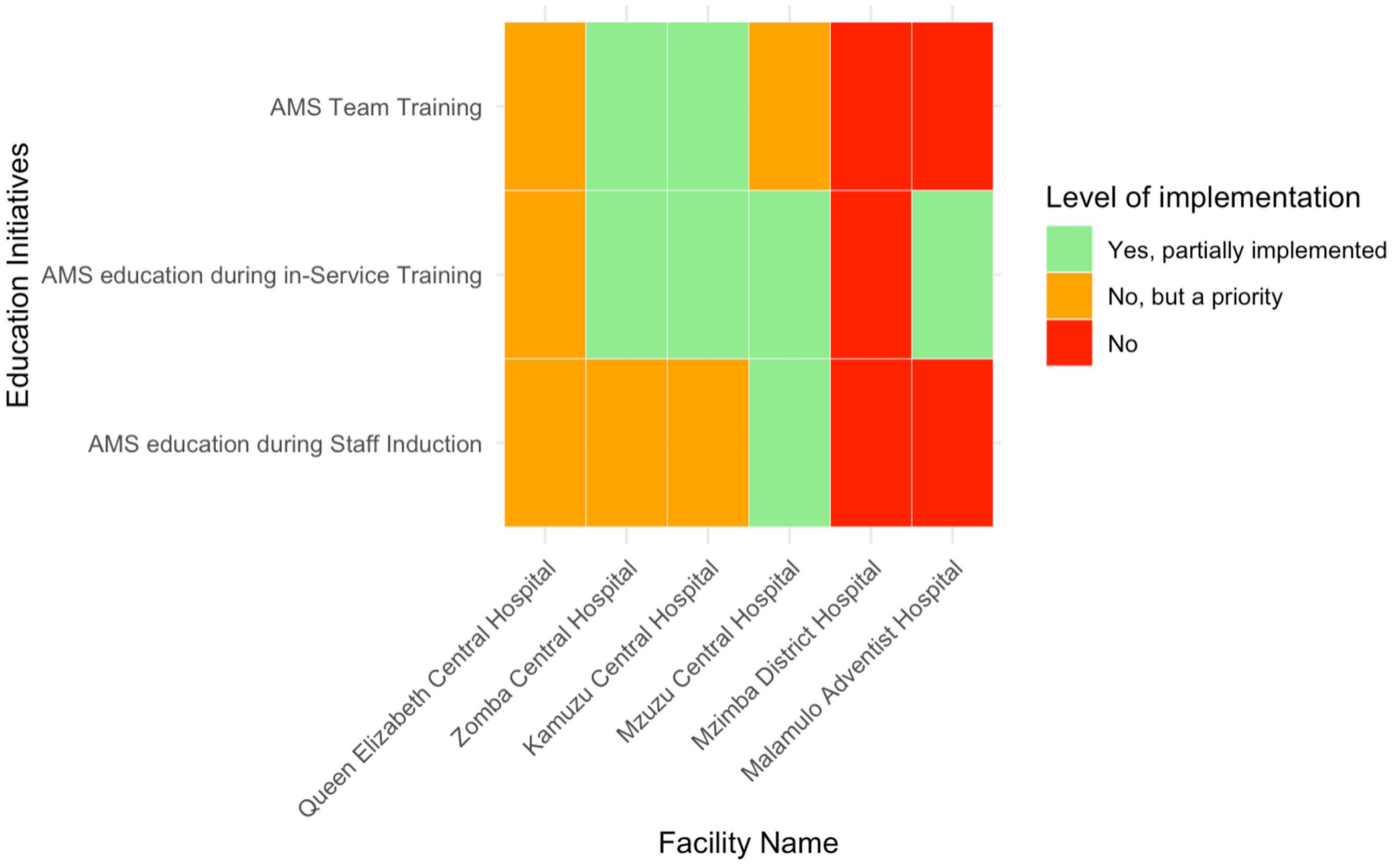
Heatmap of the level of implementation of AMS education initiatives in six Malawian hospitals.

Education initiatives focused on the inclusion of AMS programs in staff induction training and continuous professional development. AMS education as part of staff induction was inconsistently implemented across the six facilities (Figure 5). Only Mzuzu Central Hospital—representing16.7% (*n* = 1/6) of the total—reported partially integrated AMS education initiatives during staff induction, while the other facilities had either not started or prioritised it for future implementation. In-service AMS training was more commonly addressed, with partial implementation in four hospitals (66.7%, *n* = 4/6) (MCH, KCH, ZCH, and QECH). However, MDH and QECH had not initiated this training, although QECH had recognised it as a priority. Dedicated AMS team training was partially implemented in KCH, ZCH, and MCH, but the other hospitals had neither started nor prioritised this initiative.

### Monitoring and surveillance activities within AMS programs

**Figure 6:**
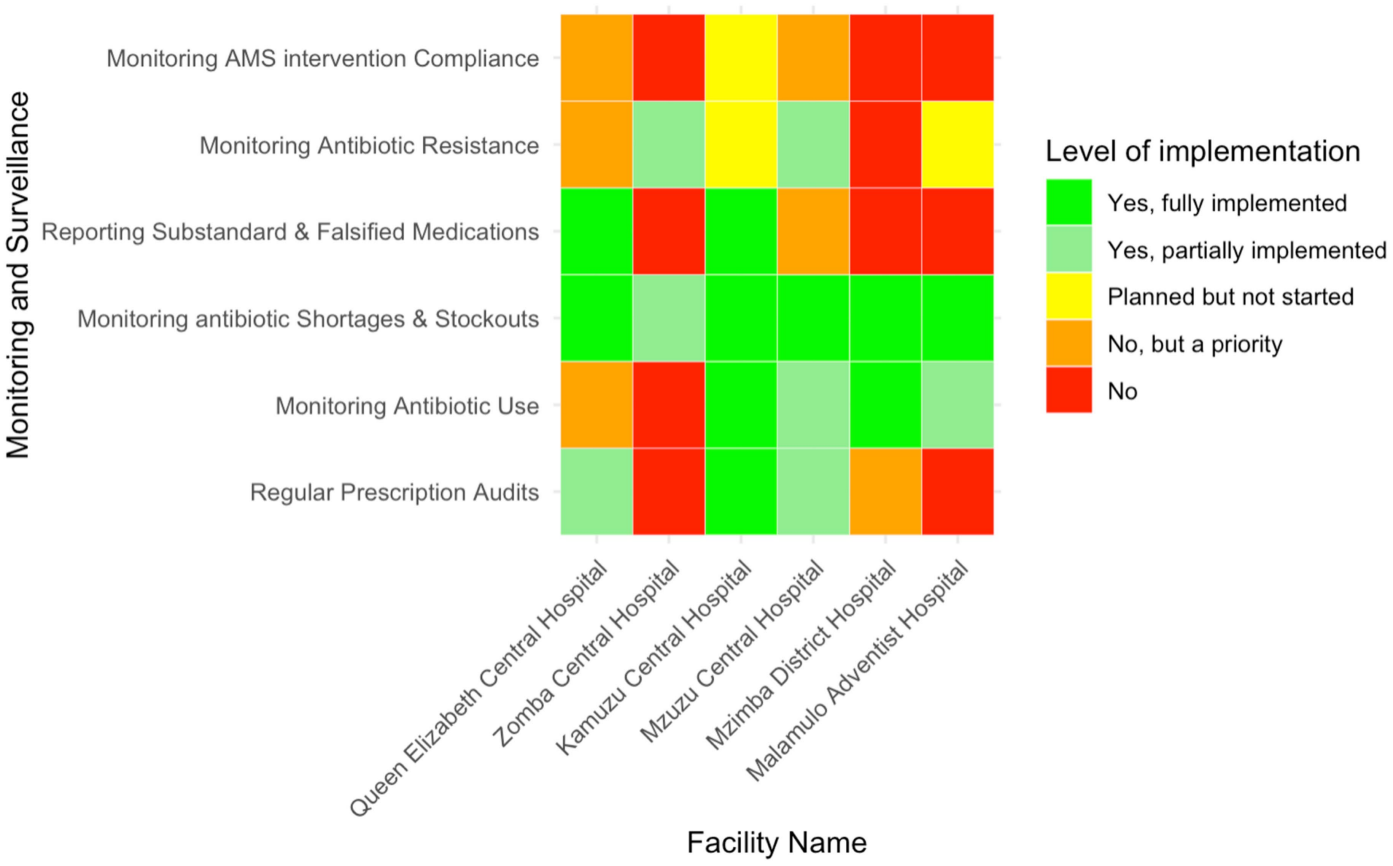
Heatmap showing the level of implementation of monitoring and surveillance activities related to AMS in six Malawian hospitals.

The implementation of regular prescription audits varied across the audited facilities (Figure 6). KCH, QECH, and MCH reported conducting regular audits −50% (*n* = 3/6)—though the latter two had only partially implemented this process. ZCH, MAH, and MDH did not perform any prescription audits, but MDH had recognised the importance of these audits and planned to implement them in the future. Similarly, monitoring systems for antibiotic use were in place at KCH, MDH, MAH, and MCH, representing 66.7% (*n* = 4/6) of the audited facilities, though MAH and MCH had only partially implemented them. ZCH and QECH had no monitoring systems, but QECH had identified it as a future priority.

Most hospitals had implemented monitoring for drug shortages and stockouts, though ZCH had only partially done so. Only KCH and QECH, representing 33.3% (*n* = 2/6), fully implemented the reporting of substandard or falsified medications, while the remaining hospitals did not. Monitoring antibiotic resistance was partially implemented at MCH and ZCH and was planned for future implementation at MAH and KCH. MDH and QECH did not monitor antibiotic resistance, though QECH recognised it as a priority. No facility monitored compliance with AMS interventions, although KCH had plans to start, and QECH and MCH acknowledged its importance for future implementation.

### Reporting activities in antimicrobial stewardship implementation

**Figure 7:**
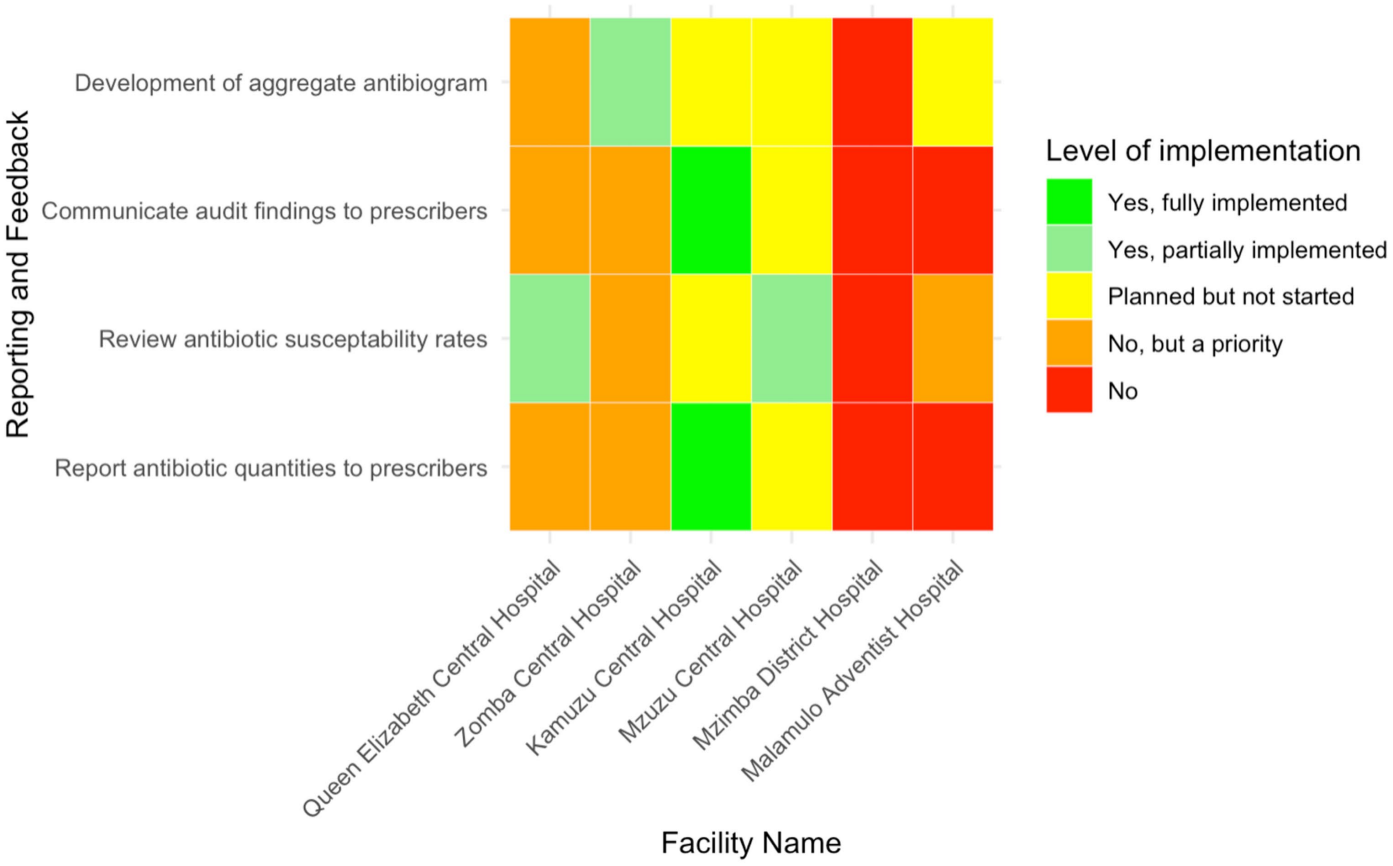
Heatmap presenting the level of implementation of reporting and feedback mechanisms related to AMS in six Malawian hospitals.

Kamuzu Central Hospital was the only facility, representing 16.7% (*n* = 1/6) of the facilities, that had reported to fully implement the practice of reporting antibiotic quantities to prescribers (Figure 7). The other hospitals had not yet adopted this practice. Zomba Central Hospital and Queen Elizabeth Central Hospital identified this activity as a priority for future implementation, while Mzuzu Central Hospital had plans in place but had not yet started. In terms of reviewing antibiotic susceptibility patterns, only MCH and QECH had partially implemented this measure, representing 33.3% of the facilities. ZCH and Malamulo Adventist Hospital prioritised it for future implementation, while KCH had plans to start but had not yet begun.

Regarding the communication of audit findings to prescribers, KCH was the only hospital that reported to fully implement this practice, representing 16.7% (*n* = 1/6) of the total. ZCH and QECH prioritised this for future implementation, while MDH and MAH had not yet communicated audit results to prescribers. Additionally, ZCH was the only facility that had partially developed aggregate antibiograms, while MAH, MCH, and KCH had plans to develop them but had not yet started. QECH also prioritised the development of aggregate antibiograms for future implementation.

### Total AMS implementation score

Table 1 outlines the AMS implementation assessment across the enrolled hospitals. Kamuzu Central Hospital achieved the highest total AMS score (79%, *n* = 129/164), followed by Mzuzu Central Hospital (69%, *n* = 113/164). Conversely, Mzimba District Hospital recorded the lowest AMS score (24%, *n* = 40/164). Queen Elizabeth Central Hospital (53%, *n* = 87/164), Malamulo Adventist Hospital (38%, *n* = 63/164,), and Zomba Central Hospital (30%, *n* = 49/164,) demonstrated moderate levels of AMS implementation. Further breakdown of the scores for each AMS core element are outlined in the Supplementary file.

**Table 1:** Antimicrobial stewardship implementation scores across healthcare facilities.

Facility name	Healthcare level	Total AMS score	Percentage (%)
Kamuzu Central Hospital	Tertiary	129/164	79
Mzuzu Central Hospital	Tertiary	113/164	69
Queen Elizabeth Central Hospital	Tertiary	87/164	53
Malamulo Adventist Hospital	Secondary	63/164	38
Zomba Central Hospital	Tertiary	49/164	30
Mzimba District Hospital	Secondary	40/164	24

The AMS score is derived from key stewardship indicators, with a maximum possible score of 164.

## Discussion

This evaluation of antimicrobial stewardship implementation across Malawi’s AMR sentinel hospitals provides critical insight into the early progress and persistent challenges of the national AMS program. Since the endorsement of the Malawian National AMR strategy in 2017 (16), which outlined strategies to optimise antibiotic use, this stands the first baseline situational analysis of its kind in Malawi. Our work fills a significant evidence gap and establishes a benchmark for future interventions and policy planning by employing a validated tool developed by the WHO.

AMS interventions centre on leadership commitment by facility management teams. Committed leaders are pivotal to the advancement and sustainability of AMS interventions, and this has been well-documented. For example, Steinmann, Lehnick (17) emphasised the significance of an empowered leadership style in AMS implementation, demonstrating a corresponding reduction in hospital-acquired infections and preventing the transmission of methicillin-resistant *Staphylococcus aureus* (MRSA) infections in a Swiss children’s hospital. While it is encouraging that AMS was recognised as a priority by all participating hospitals, the inconsistent translation of this recognition into structured plans, resource allocation, and monitoring systems reflects a broader issue of implementation. These gaps highlight the need for facility-level leadership to move beyond passive endorsement toward tangible institutional integration of AMS. Ideally, AMS should be included in facility strategic plans and in staff job descriptions to ensure healthcare professionals have dedicated time for stewardship activities (8). This not the case for Malawi, as demonstrated in this study; however, countries such as the United Kingdom and Canada mandate dedicated stewardship teams (18). However, our findings are similar to those reported in Zambia, where 50% (4/8) of evaluated facilities scored below average on leadership commitment to AMS activities (19), this alludes to a regional pattern worth further exploration.

The WHO recommends that healthcare facilities should establish clear AMS interventions to optimise antibiotic use and reduce resistance (8, 20). Multidisciplinary ward rounds, for example, have been shown to improve antibiotic use, enhance patient outcomes, and reduce healthcare costs (21, 22). In the United Kingdom, introducing multidisciplinary ward rounds in two Scottish hospitals improved therapeutic management (escalation, de-escalation and intravenous-to-oral switching) in up to 69.2% of antimicrobial prescriptions, leading to cost savings of up to 24.9% (21). A similar pattern was demonstrated in QECH, where a 2016 multidisciplinary ward round resulted in a 26.5% reduction in Third-Generation Cephalosporin prescriptions, saving approximately US$15,000 annually, with no change in the case-fatality rate (23). The limited uptake of these high-impact practices across audited facilities suggests the need to tailor interventions to local capacity, without compromising core stewardship principles. Evidence from regional settings like Zambia (19) and South Africa (24) reinforces the importance of adapting AMS actions to context—ensuring they are feasible, sustainable, and aligned with health system realities.

Education and training is a major area requiring strategic investment for successful AMS programs in Africa (24). The presence of AMS training within facility discourse is promising, but sporadic implementation and limited integration into institutional frameworks limit its potential impact. These findings align with a recent WHO AFRO regional assessment (25), which reported significant gaps in AMS-related education and training across the African continent. According to this assessment, only 45.2% of countries had incorporated AMS principles into the curricula for healthcare professionals, and just 35.5% provided government-supported in-service AMS training specifically targeting AMS teams. Broader AMS training for general healthcare workers was available in fewer than half of the countries surveyed (14/31), and only 25.8% had any structured education on rational antibiotic use or infection prevention and control at the basic or secondary school level. Alarmingly, just three countries (9.7%) offered government incentives, staffing support, or accreditation standards to promote AMS education within healthcare facilities. These findings underscore the need for systemic investment in AMS education—both within professional training pipelines and through structured in-service opportunities. In the Malawian context, where AMS programs are still emerging, sustained educational interventions must be embedded in institutional training plans and supported by national policy.

This study is subject to several limitations. First, the assessment only included facilities that confirmed AMS activity implementation. While this was a cost-effective approach for identifying engaged facilities, it may have excluded others that are implementing AMS without formal recognition. Second, the assessment focused on facilities within Malawi’s national AMR surveillance network, potentially excluding a broader spectrum of hospitals with differing AMS capacities. This compromises the generalisability of these findings. Third, the accuracy of the findings may have been influenced by recall bias, as responses were provided through group consensus. Additionally, the self-assessment nature of the audit may have introduced social desirability bias, with some teams potentially overestimating their AMS performance. Lastly, the use of a structured nature of the WHO toolkit limited exploration of underlying contextual drivers of implementation challenges. Future research should prioritize in-depth qualitative approaches to explore these root causes more comprehensively.

## Conclusion

Three key gaps, in the early implementation of AMS programs in Malawi’s public hospitals have been identified: limited leadership commitment, inconsistent AMS ward rounds, and insufficient education for healthcare workers. Addressing these challenges will require targeted strategies to strengthen leadership accountability, institutionalize routine AMS actions, and build ongoing capacity through structured training. These actions are essential to ensure meaningful progress in Malawi’s national AMS program.

## Data Availability

The datasets presented in this study can be found in online repositories. The names of the repository/repositories and accession number(s) can be found at: https://data.mendeley.com/datasets/8mxs2rmfyg/1.

## Footnotes

^1^ https://data.mendeley.com/datasets/8mxs2rmfyg/1
