# Supplementary material for: A baseline assessment of antimicrobial stewardship core element implementation in selected public hospitals in Malawi: findings from the 2023 National Program Audit
Source: Front Public Health. 2025 Jun 12;13:1588778. doi: 10.3389/fpubh.2025.1588778 (PMC12198209; doi:10.3389/fpubh.2025.1588778)
Supplement: Supplementary file 1 [file Data_Sheet_1.PDF]

MALAWI HEALTHCARE FACILITY CORE ELEMENT ASSESSMENT FOR AMS_v1

Facility Demographics

Name of faciltiy completing the AMS assessment tool

- [ ] Mzuzu Central Hospital
- [ ] Mzimba District Hospital
- [ ] Kamuzu Central Hospital
- [ ] Mangochi District Hospital
- [ ] Queen Elizabeth Central Hospital
- [ ] Zomba Central Hospital
- [ ] Malamulo Adventist Hospital

Location of facility completing the AMS assessment tool

- [ ] Northern
- [ ] Central
- [ ] Southern

Facility level of care

- [ ] Primary
- [ ] Secondary
- [ ] Tertiary

Coordinates of Hospital

latitude (x.y °)

____

longitude (x.y °)

____

altitude (m)

____

accuracy (m)

____

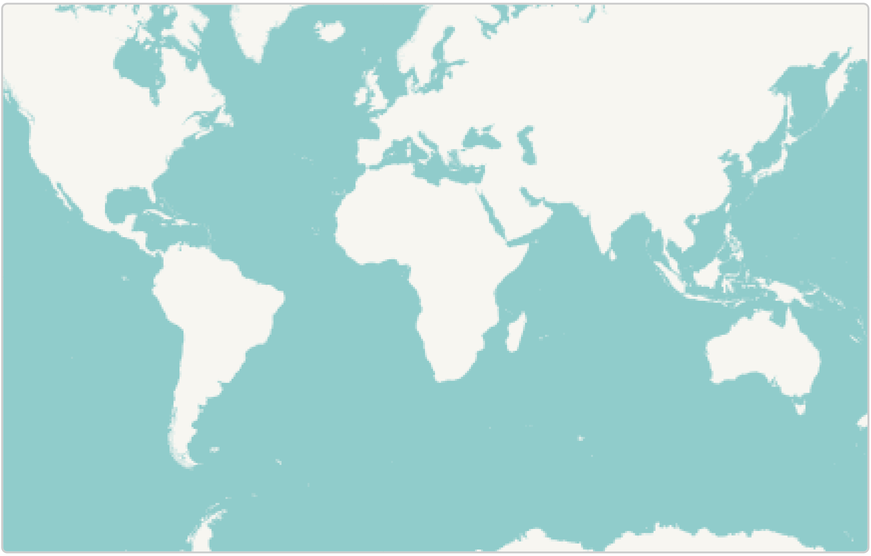

Leadership Commitment

Is Antimicrobial Stewardship (AMS) identified as a priority by the health-care facility management/leadership?

- [ ] No
- [ ] No, but a priority
- [ ] Planned but not started
- [ ] Yes, partially implemented
- [ ] Yes, fully implemented

Are AMS activities included in health-care facility annual plans with key performance indicators?

- [ ] No
- [ ] No, but a priority
- [ ] Planned but not started
- [ ] Yes, partially implemented
- [ ] Yes, fully implemented

Has the health-care facility management allocated human and financial resources to initiate AMS activities?

- [ ] No
- [ ] No, but a priority
- [ ] Planned but not started
- [ ] Yes, partially implemented
- [ ] Yes, fully implemented

Is there a health-care facility action plan in place that prioritizes AMS activities?

- [ ] No
- [ ] No, but a priority
- [ ] Planned but not started
- [ ] Yes, partially implemented
- [ ] Yes, fully implemented

Is there a mechanism to regularly monitor and measure the implementation of AMS activities?

- [ ] No
- [ ] No, but a priority
- [ ] Planned but not started
- [ ] Yes, partially implemented
- [ ] Yes, fully implemented

Is there dedicated financial support for the health-care facility AMS action plan?

- [ ] No
- [ ] No, but a priority
- [ ] Planned but not started
- [ ] Yes, partially implemented
- [ ] Yes, fully implemented

Has a budget (e.g. annual) for the implementation of the health-care facility AMS action plan been developed?

- [ ] No
- [ ] No, but a priority
- [ ] Planned but not started
- [ ] Yes, partially implemented
- [ ] Yes, fully implemented

Accountability and Responsibility

Is there a multidisciplinary AMS committee leadership in the health-care facility with clear terms of reference?

- [ ] No
- [ ] No, but a priority
- [ ] Planned but not started
- [ ] Yes, partially implemented
- [ ] Yes, fully implemented
- [ ] Not applicable

Does the AMS committee/other relevant committee meet on a regular basis (minimum monthly or quarterly)?

- [ ] No
- [ ] No, but a priority
- [ ] Planned but not started
- [ ] Yes, partially implemented
- [ ] Yes, fully implemented
- [ ] Not applicable

Is there a dedicated AMS leader/champion identified for the health-care facility?

- [ ] No
- [ ] No, but a priority
- [ ] Planned but not started
- [ ] Yes, partially implemented
- [ ] Yes, fully implemented
- [ ] Not applicable

Does the team leader or champion have dedicated staff time for AMS activity in their TOR/job description?

- [ ] No
- [ ] No, but a priority
- [ ] Planned but not started
- [ ] Yes, partially implemented
- [ ] Yes, fully implemented
- [ ] Not applicable

Is there an AMS team with clear terms of reference?

- [ ] No
- [ ] No, but a priority
- [ ] Planned but not started
- [ ] Yes, partially implemented
- [ ] Yes, fully implemented
- [ ] Not applicable

Does the AMS team meet on a regular basis?

- [ ] No
- [ ] No, but a priority
- [ ] Planned but not started
- [ ] Yes, partially implemented
- [ ] Yes, fully implemented
- [ ] Not applicable

Are other health-care professionals apart from AMS team involved in AMS activities?

- [ ] No
- [ ] No, but a priority
- [ ] Planned but not started
- [ ] Yes, partially implemented
- [ ] Yes, fully implemented
- [ ] Not applicable

Does the AMS committee/team collaborate with the IPC team at the health-care facility-if the team is separate?

- [ ] No
- [ ] No, but a priority
- [ ] Planned but not started
- [ ] Yes, partially implemented
- [ ] Yes, fully implemented
- [ ] Not applicable

Does the AMS team/committee produce regular (descriptive) activity reports on the implementation of the AMS programme?

- [ ] No
- [ ] No, but a priority
- [ ] Planned but not started
- [ ] Yes, partially implemented
- [ ] Yes, fully implemented
- [ ] Not applicable

Is the AMS activity report disseminated to the facility management, other health-care facility team members and appropraite national authority?

- [ ] No
- [ ] No, but a priority
- [ ] Planned but not started
- [ ] Yes, partially implemented
- [ ] Yes, fully implemented
- [ ] Not applicable

AMS actions

Is there a standard treatment guideline at the health-care facility?

- [ ] No
- [ ] No, but a priority
- [ ] Planned but not started
- [ ] Yes, partially implemented
- [ ] Yes, fully implemented

Are the guidelines reviewed and updated periodically based on the availability of new evidence?

- [ ] No
- [ ] No, but a priority
- [ ] Planned but not started
- [ ] Yes, partially implemented
- [ ] Yes, fully implemented

Is there a regular review/audit of specified antibiotic therapy or clinical conditions at the health-care facility?

- [ ] No
- [ ] No, but a priority
- [ ] Planned but not started
- [ ] Yes, partially implemented
- [ ] Yes, fully implemented

Is the advice/feedback from AMS teams easily accessible/available to prescribers?

- [ ] No
- [ ] No, but a priority
- [ ] Planned but not started
- [ ] Yes, partially implemented
- [ ] Yes, fully implemented

Does the AMS team conduct regular ward rounds and other AMS interventions in selected departments in the health-care facility?

- [ ] No
- [ ] No, but a priority
- [ ] Planned but not started
- [ ] Yes, partially implemented
- [ ] Yes, fully implemented

Does the health-care facility have a formulary/ list of approved antibiotics for use based on the national formulary?

- [ ] No
- [ ] No, but a priority
- [ ] Planned but not started
- [ ] Yes, partially implemented
- [ ] Yes, fully implemented

"Does the health-care facility formulary specify lists of restricted antibiotics that require approval by a designated team or person (pre-authorization)? "

- [ ] No
- [ ] No, but a priority
- [ ] Planned but not started
- [ ] Yes, partially implemented
- [ ] Yes, fully implemented

Does the health-care facility have access to laboratory and imaging services (on-site or off-site) that can be used to support AMS interventions?

- [ ] No
- [ ] No, but a priority
- [ ] Planned but not started
- [ ] Yes, partially implemented
- [ ] Yes, fully implemented

Are there information technology services, tally cards or other inventory control tools available that can be used to support data gathering to support AMS activities?

- [ ] No
- [ ] No, but a priority
- [ ] Planned but not started
- [ ] Yes, partially implemented
- [ ] Yes, fully implemented

Is there standardized prescription charts, medical records/patient folders and transfer notes to support treatment and AMS activities?

- [ ] No
- [ ] No, but a priority
- [ ] Planned but not started
- [ ] Yes, partially implemented
- [ ] Yes, fully implemented

Does the health-care facility have a written policy that requires prescribers to document the indication and antibiotics prescribed in a prescription chart/ medical records?

- [ ] No
- [ ] No, but a priority
- [ ] Planned but not started
- [ ] Yes, partially implemented
- [ ] Yes, fully implemented

Education and Training

Does the health-care facility include AMS program such as optimizing antibiotic prescribing, dispensing and administration in the staff induction training?

- [ ] No
- [ ] No, but a priority
- [ ] Planned but not started
- [ ] Yes, partially implemented
- [ ] Yes, fully implemented

Does the health-care facility offer continue in-service training or continuous professional development on AMS, IPC to staff?

- [ ] No
- [ ] No, but a priority
- [ ] Planned but not started
- [ ] Yes, partially implemented
- [ ] Yes, fully implemented

Does the health-care facility/ ensure training for AMS team on antimicrobial stewardship/ infection prevention and control

- [ ] No
- [ ] No, but a priority
- [ ] Planned but not started
- [ ] Yes, partially implemented
- [ ] Yes, fully implemented

Monitoring and surveillance

"Are regular prescription audits, point prevalence surveys to assess the appropriateness of antibiotic prescribing undertaking at the facility by AMS committee or relevant team? "

- [ ] No
- [ ] No, but a priority
- [ ] Planned but not started
- [ ] Yes, partially implemented
- [ ] Yes, fully implemented

Does the health-care facility regularly monitor the quantity and types of antibiotic use (purchased/prescribed/dispensed)?

- [ ] No
- [ ] No, but a priority
- [ ] Planned but not started
- [ ] Yes, partially implemented
- [ ] Yes, fully implemented

Does the health-care facility regularly monitor shortages/stockouts of essential antimicrobials?

- [ ] No
- [ ] No, but a priority
- [ ] Planned but not started
- [ ] Yes, partially implemented
- [ ] Yes, fully implemented

Is there a mechanism to report substandard and falsified medicines and diagnostics at the health-care facility?

- [ ] No
- [ ] No, but a priority
- [ ] Planned but not started
- [ ] Yes, partially implemented
- [ ] Yes, fully implemented

Does the AMS team regularly monitor antibiotic susceptibility and resistance rate for a range of key indicator bacteria

- [ ] No
- [ ] No, but a priority
- [ ] Planned but not started
- [ ] Yes, partially implemented
- [ ] Yes, fully implemented

Does the AMS team monitor compliance with at least one specific AMS intervention (e.g. indication captured in medical records for patients) at the health-care facility?

- [ ] No
- [ ] No, but a priority
- [ ] Planned but not started
- [ ] Yes, partially implemented
- [ ] Yes, fully implemented

Reporting Feedback within the Health-care facility

Does the AMS committee/relevant team analyze and report on the quantities of antibiotics purchased/prescribed/dispensed to prescribers and health-care facility management?

- [ ] No
- [ ] No, but a priority
- [ ] Planned but not started
- [ ] Yes, partially implemented
- [ ] Yes, fully implemented

Does the AMS committee/ relevant team review, analyze and reports on antibiotic susceptibility rates and key findings shared with prescribers?

- [ ] No
- [ ] No, but a priority
- [ ] Planned but not started
- [ ] Yes, partially implemented
- [ ] Yes, fully implemented

Does the AMS team communicate findings from audits/ reviews of the quality/appropriateness of antibiotic use to prescribers along with specific action points?

- [ ] No
- [ ] No, but a priority
- [ ] Planned but not started
- [ ] Yes, partially implemented
- [ ] Yes, fully implemented

Does the health-care facility develop and aggregate antibiogram (annex VIII, page 71 of WHO AMS toolkit) and regularly update it?

- [ ] No
- [ ] No, but a priority
- [ ] Planned but not started
- [ ] Yes, partially implemented
- [ ] Yes, fully implemented
