# Supplementary material for: A baseline assessment of antimicrobial stewardship core element implementation in selected public hospitals in Malawi: findings from the 2023 National Program Audit
Source: Front Public Health. 2025 Jun 12;13:1588778. doi: 10.3389/fpubh.2025.1588778 (PMC12198209; doi:10.3389/fpubh.2025.1588778)
Supplement: Supplementary file 4 [file Table_3.DOCX]

**Table 4 : AMS action scores across healthcare facilities.**

| **Facility Name** | **AMS action Score** | **Percentage (%)** |
| --- | --- | --- |
| Zomba Central Hospital | 14 | 32 |
| Mzimba District Hospital | 18 | 41 |
| Queen Elizabeth Central Hospital | 23 | 52 |
| Mzuzu Central Hospital | 25 | 57 |
| Malamulo Adventist Hospital | 26 | 59 |
| Kamuzu Central Hospital | 36 | 82 |
