# Supplementary material for: A baseline assessment of antimicrobial stewardship core element implementation in selected public hospitals in Malawi: findings from the 2023 National Program Audit
Source: Front Public Health. 2025 Jun 12;13:1588778. doi: 10.3389/fpubh.2025.1588778 (PMC12198209; doi:10.3389/fpubh.2025.1588778)
Supplement: Supplementary file 6 [file Table_5.DOCX]

**Table 6: AMS-specific monitoring scores across healthcare facilities in malawi.**

| **Facility Name** | **Monitoring Score** | **Percentage (%)** |
| --- | --- | --- |
| Zomba Central Hospital | 6 | 25 |
| Malamulo Adventist Hospital | 9 | 38 |
| Mzimba District Hospital | 9 | 38 |
| Queen Elizabeth Central Hospital | 14 | 58 |
| Mzuzu Central Hospital | 15 | 62 |
| Kamuzu Central Hospital | 20 | 83 |
