# Supplementary material for: A baseline assessment of antimicrobial stewardship core element implementation in selected public hospitals in Malawi: findings from the 2023 National Program Audit
Source: Front Public Health. 2025 Jun 12;13:1588778. doi: 10.3389/fpubh.2025.1588778 (PMC12198209; doi:10.3389/fpubh.2025.1588778)
Supplement: Supplementary file 7 [file Table_6.DOCX]

**Table 7: AMS reporting scores across healthcare facilities in Malawi.**

| **Facility Name** | **Reporting Score** | **Percentage (%)** |
| --- | --- | --- |
| Mzimba District Hospital | 0 | 0 |
| Malamulo Adventist Hospital | 3 | 19 |
| Queen Elizabeth Central Hospital | 6 | 38 |
| Zomba Central Hospital | 6 | 38 |
| Mzuzu Central Hospital | 9 | 56 |
| Kamuzu Central Hospital | 12 | 75 |
